# Transcriptome and Hormone Regulations Shape Drought Stress‐Dependent Fusarium Head Blight Susceptibility in Different Barley Genotypes

**DOI:** 10.1111/pce.70566

**Published:** 2026-04-30

**Authors:** Felix Hoheneder, Christina Elisabeth Steidele, Michael Gigl, Corinna Dawid, Ralph Hückelhoven

**Affiliations:** ^1^ Chair of Phytopathology, TUM School of Life Sciences Technical University of Munich Freising Weihenstephan Germany; ^2^ Junior Research Group Food Processing and Health, ZIEL Institute for Food and Health Technical University of Munich Freising Weihenstephan Germany; ^3^ Chair of Chemosensory Food Systems, TUM School of Life Sciences Technical University of Munich Freising Weihenstephan Germany; ^4^ Leibniz Institute for Food Systems Biology at the Technical University of Munich Freising Weihenstephan Germany

**Keywords:** abscisic acid, barley, drought, Fusarium Head Blight, phytohormones, stress combination, transcriptomics

## Abstract

Little is known about regulatory mechanisms that crop plants use to respond to combinations of abiotic and biotic stress. We analysed four barley genotypes under simultaneous *Fusarium culmorum* infection and drought stress by phenotyping for Fusarium Head Blight (FHB) disease, drought stress responses, hormone profiling and transcriptome analysis. FHB severity was host genotype‐dependent, with moderately resistant cultivars Avalon and Barke showing increased FHB under drought, while drought did not further increase FHB severity in highly susceptible Morex and Palmella Blue. Transcriptome analysis revealed largely additive effects of single stresses, with drought‐dominated regulation with increasing drought severity. Co‐expression analysis connected abscisic acid and auxin to gene expression modules functionally enriched with stress‐specific physiological responses. Stress‐response genes, uniformly expressed across genotypes, were linked to pathogen defence, detoxification and drought adaptation, whereas a cluster of hundreds of moderately *Fusarium*‐responsive genes was limited in up‐regulation under combined stress, possibly explaining enhanced FHB severity under drought. A multiple linear regression model accurately predicted combined stress expression from single stress responses, demonstrating that gene regulation under combined drought and *Fusarium* stress is largely driven by additive effects of individual stresses.

## Summary statement

FHB severity in spring barley under drought is genotype‐dependent, with moderately resistant cultivars showing increased FHB while highly susceptible ones remained unaffected. Transcriptome analysis showed largely additive stress effects with drought‐dominated regulation as severity increases.

## 1 Introduction

During the preparation of this study, 2024 marked a significant milestone, with the global average temperature exceeding the pre‐industrial baseline by 1.5°C for one full year for the first time (Copernicus Climate Change Service 2025). This threshold is widely regarded as a critical limit for irreversible environmental damage (Doelman et al. 2019). Europe is warming faster than the global average (van der Schrier et al. 2013) experiencing more frequent droughts (Hanel et al. 2018; Spinoni et al. 2018), which significantly threaten cereal production (Naumann et al. 2015; Brás et al. 2021; Martin et al. 2025). Simultaneously, climate change is intensifying plant disease dynamics by altering pathogen abundance, distribution and host susceptibility (Chakraborty and Newton 2011; Delgado‐Baquerizo et al. 2020; Martínez et al. 2022). Rising temperatures, air humidity and atmospheric CO₂ levels are driving changes in pathogen abundance, geographic distribution and host susceptibility, creating increasingly complex stress environments and weather events. For *Fusarium* spp., climate‐driven shifts in species and chemotypes are changing mycotoxin profiles, posing risks to food safety (Nnadi and Carter 2021; Johns et al. 2022).

While the individual impacts of drought and pathogens on plants are well studied, their simultaneous occurrence triggers complex and often unpredictable plant responses (Gupta et al. 2016; Zandalinas et al. 2021). These stress interactions involve signal cross‐talk, resource trade‐offs and altered defence strategies (Karasov et al. 2017; Leisner et al. 2023). Even mild stresses, when combined, can have disproportionate effects on plant health and yield, depending on the timing, sequence and dominance of each stressor (Pandey and Senthil‐Kumar 2019; Zandalinas et al. 2021). Abscisic acid (ABA) is the primary drought‐signalling molecule regulating stress adaptation, gene expression and growth. ABA induces transcription factors involved in drought and pathogen stress (Yao et al. 2020), whereas salicylic acid (SA) and jasmonic acid (JA)/ethylene pathways are central in defence against *Fusarium* infections and strongly linked to host physiology in cereals (Wang et al. 2018; Ding et al. 2011). Ethylene, auxin and ABA are associated with FHB susceptibility (Chen et al. 2009; Xiao et al. 2013; Brauer et al. 2019; Qi et al. 2016; Buhrow et al. 2021), whereas SA, JA and gibberellic acid appear involved in FHB resistance (Buhrow et al. 2021; Ding et al. 2011; Makandar et al. 2012; Sun et al. 2016). These complex physiological and molecular responses to multiple stresses remain poorly understood in cereals.

In cereals, drought and heat alter host susceptibility and pathogen dynamics of *Fusarium* spp., the causal agents of Fusarium Head Blight (FHB) (Liu and Liu 2016; Hoheneder et al. 2023; Martínez et al. 2022), making it difficult to predict disease pressure and complicating control strategies (Lahlali et al. 2024). Moreover, responses of wheat and barley to combined stresses, such as drought, heat and *Fusarium* infection, are highly complex and genotype‐dependent, triggering unique transcriptional profiles, with drought often dominating and modulating defence‐ and stress‐related responses (Mikołajczak et al. 2022; Su et al. 2024; Hoheneder et al. 2023). Notably, wheat and barley show strongly related transcriptional responses between genes associated with drought tolerance and those responding to Fusarium crown rot infection (Su, Powell, et al. 2021). These findings highlight potential crosstalk between abiotic and biotic pathways, emphasizing that integrated stress signalling could shape defence and abiotic stress tolerance in cereals. This is particularly concerning in cereals, where *Fusarium* spp. reduce yield and contaminate grain with harmful mycotoxins (Wegulo et al. 2015). Despite these evident risks, crop breeding efforts have rather neglected combined stress responses, likely due to their complexity.

Here, we investigated how simultaneous *Fusarium* infection and drought stress affect FHB severity, stress‐related gene expression and hormonal regulation in spring barley. We reveal a genotype‐dependent increase in FHB severity under drought, accompanied by strong additive transcriptomic responses. Notably, we identified core sets of differentially expressed genes shared across individual and combined stress conditions, and used a linear regression model to predict gene expression under combined stress from single stress profiles.

## 2 Materials and Methods

### 2.1 Greenhouse Experiment

Four spring barley genotypes (Avalon, Barke, Morex and Palmella Blue) were cultivated under controlled glasshouse conditions at the Greenhouses and Phytochambers Unit of the Plant Technology Center, Technical University of Munich. These genotypes differ in quantitative resistance to FHB determined in field (Hoheneder et al. 2022) and greenhouse experiments (Hoheneder et al. 2023), with Avalon tested in preliminary field and greenhouse experiments.

Five grains were sown per 3‐L pot containing 900 g of peat substrate (Einheitserde C700, Stender, Germany). Eighteeen pots per genotype and treatment group were cultivated under long‐day conditions (16 h light), at 18°C day/16°C night and 60% relative humidity.

Drought stress was applied to half of the pots starting from the mid‐flowering stage (growth stage 65) by a stop of automatic daily watering. These drought‐exposed pots were weighed to monitor water loss at 0, 1, 2, 4, and 8 days post‐watering cessation (dpw), and were manually supplemented with 75 mL of water at 4 dpw and 50 mL at 8 dpw. Severe drought was achieved by reducing the relative pot weight to 40% by 8 dpw until growth stage 90 (Supporting Information S1: Figure S1). At mid‐flowering, half of the irrigated plants and half of the drought‐designated pots were sprayed till run‐off with either a *Fusarium culmorum* (Fc) spore solution or a mock solution as described in detail by Hoheneder et al. (2023), resulting in the four treatments: WM (watered+mock), WFc (watered+infected), DM (drought+mock) and DFc (drought+infected). At 2, 4 and 7 dpi, four pots per treatment were randomly selected, and individual spikes (four per sample) were harvested and flash‐frozen in liquid nitrogen, obtaining four biological replicates.

### 2.2 Preparation of *F. culmorum* Inoculum and Mock Solution

The *F. culmorum* inoculum (isolates Fc002, Fc03 and Fc06; culture collection, Chair of Phytopathology, Technical University of Munich) was cultured as outlined by Linkmeyer et al. (2013). The spore solution was prepared as described by Hoheneder et al. (2023).

### 2.3 Phytohormone and Metabolite Quantification

Spike samples (150 mg ground tissue, identical to those used for DNA/RNA extraction) were used to quantify ABA, abscisic acid glucoside (ABA‐Glc), phaseic acid (PA), dihydrophaseic acid (DPA), auxin (IAA), JA, SA, salicylic acid glucoside (SA‐Glc) and the osmolyte proline (Chaudhary et al. 2020). Samples were supplemented with internal standards, extracted in ethyl acetate and the filtered supernatant was analysed by LC‐MS/MS in multiple reaction‐monitoring mode. Data were acquired using Analyst 1.6.3 (Sciex, Darmstadt, Germany).

Gaseous ethylene (ET) production in spikes was measured in four replicates per treatment at 2, 4 and 7 dpi. Spikes were sampled in glass vials containing 1 mL of sterile water to prevent the spikes from desiccation. The vials were closed with gas‐tight rubber lids and incubated for 5 h under daylight at room temperature. One milliliter of the headspace was injected into a gas chromatograph (Varian Aerograph 3300, Shimadzu, Japan) equipped with a deactivated aluminium oxide column and combined with an Integrator (C‐R6A Chromatopac, Shimadzu, Japan). ET amounts (pmol/mL air) were calculated by dividing peak areas by 214 (internal calibration: 1 mL pure ethylene gas = 214 pmol at 1 bar) (Von Kruedener et al. 1995) and normalized to spike fresh weight.

### 2.4 Area Under the Curve (AUC) Analyses

Phytohormone and proline quantities were used to calculate the AUC (4–7 dpi) (Shaner and Finney 1977). Similarly, area under the disease progression curve (AUDPC) values were calculated for FHB symptoms and fungal DNA in infected and drought‐plus‐infected samples. Log_2_ fold changes of AUCs were computed relative to watered mock‐treated plants (WFc, DM, DFc). Hierarchical clustering grouped phytohormone responses across cultivars and treatments using MultiExperiment Viewer 4.9.0 (Howe et al. 2011).

### 2.5 Measurement of Chlorophyll Contents and Stomatal Conductance

Physiological responses were assessed by measuring chlorophyll content and stomatal conductance of upper leaves (flag leaf [F] and flag leaf‐1 [F‐1]). Chlorophyll was recorded as SPAD values on 18 leaves per stage at 2, 4 and 7 dpw using a SPAD502 meter (Konica Minolta, Japan). Stomatal conductance was measured on 15 leaves per stage at 3, 5 and 8 dpw using an SC‐1 Leaf Porometer (Meter Group, Germany).

### 2.6 DNA and RNA Extraction

Spike samples were ground in liquid nitrogen for DNA extraction following Fraaije et al. (1999). DNA was diluted to 20 ng/μL. RNA was extracted using the GeneMATRIX Universal RNA Purification Kit (EURx, Poland) with DNA digestion, quantified with a Qubit 2.0 fluorometer, and diluted to 100 ng/µL.

### 2.7 Quantification of Fungal DNA

*F. culmorum* and barley DNA in spike samples were quantified using the qPCR protocol and species‐specific primers from Nicolaisen et al. (2009) as described in detail by Hoheneder et al. (2023).

### 2.8 Library Preparation and 3′RNA‐Sequencing

Libraries for 3′‐RNA sequencing were prepared using the QuantSeq 3′mRNA‐Seq Library Prep Kit (Lexogen, Austria) following the manufacturer's protocol (Moll et al. 2014). Library concentrations were quantified with a Qubit 2.0 and Qubit DNA HS Assay, and pool concentrations were determined by qPCR. Library integrity and fragment size were assessed with an Agilent 2010 Bioanalyzer (HS DNA Assay). Sequencing was performed on an Illumina NovaSeq. 6000.

### 2.9 Read Quality Check, Trimming, Mapping and Annotation

3'mRNAseq reads were processed with nf‐core/rnaseq (3.17.0) using Nextflow (24.04.4) (Ewels et al. 2020). The quality of the raw reads was checked using FastQC (0.12.1), adapter trimming and filtering were performed with Trim Galore (v0.6.10) and Cutadapt (4.9). STAR (2.7.11b) was used for read alignment against the barley reference genome Morex v3 (Mascher et al. 2021) merged with the *F. culmorum* genome FusCulm_01 (GenBank assembly GCA_003033665.1) and Salmon (1.10.3) for transcript quantification. The 3′UTR regions of each gene were extended by 3 kb or until the next gene to compensate for incomplete 3′UTR annotation and mapping rates of 3′‐end biased 3′mRNAseq reads.

### 2.10 Differential Gene Expression Analysis and WGCNA

Differential gene expression analysis was performed with edgeR v4.0.16 (Robinson et al. 2010). For each time point (4, 7 dpi), differential expression was calculated as variety_condition – variety_watered‐mock, where variety = Avalon, Barke, Morex or Palmella Blue and condition = watered‐infected, drought‐mock or drought‐infected, yielding 15,094 DEGs. Significant DEGs were filtered by FDR‐corrected *p* < 0.01 and log_2_FC (log_2_FC < 0: down‐regulated; log_2_FC > 0: up‐regulated) (Supporting Information S1: Figure S2). Co‐expression analysis was performed using the WGCNA R package (Langfelder and Horvath 2008, 2012) on 6558 significant DEGs, using their normalized counts per million. Co‐expressed gene modules were identified and correlated with phenotypic traits (quantified phytohormones, proline, fungal DNA contents and FHB symptoms; see Supporting Information S4: Dataset_P: Phenotypic data). Network construction used the following parameters: blockwiseModules(mydatExpr, power = 8, TOMType = ‘signed’, minModuleSize = 30, reassignThreshold = 0, mergeCutHeight = 0.25, numericLabels = TRUE, pamRespectsDendro = FALSE, saveTOMs = TRUE, saveTOMFileBase = ‘mydatTOM’, verbose = 3).

### 2.11 DEG Cluster Analysis

DEGs were clustered by expression pattern across treatments with a self‐organizing tree algorithm (SOTA) in MultiExperiment Viewer v4.9.0 (Howe et al. 2011) using 6558 significant DEGs (FDR‐corrected *p* < 0.01; |log_2_FC | > 1) in at least one genotype.

### 2.12 Gene Ontology (GO) Enrichment Analysis

GO enrichment analysis was performed using g:Profiler (Kolberg et al. 2023) with default parameters, except term size of at least five genes. The most enriched biological process (BP), molecular function (MF) and cellular component (CC) were visualized using ‘ggplot2’ and ‘dplyr’ in R Studio to display the most significant GO terms (based on the −log10 adjusted *p* value), the respective gene counts (DEGs per GO term) and gene ratios (DEGs per GO term/total DEGs).

### 2.13 Similarity Score Analysis

To compare gene sets in single or combined stress, a Similarity score (Jaccard index) was calculated following Tan et al. (2023) as the ratio of the intersection to the union of two DEG sets. Therefore, Venn diagrams for up‐ and down‐regulated DEGs per treatment, genotype and time point were generated (Supporting Information S1: Figure S3) using Venny 2.1.0.

### 2.14 Similarity of Gene Expression Profiles Under Single and Combined Stress

To assess similarity of gene expression profiles under combined stress (log_2_FC DFc) versus individual stresses (log_2_FC WFc, log_2_FC DM), absolute differences |log_2_FC DFc − log_2_FC WFc| and |log_2_FC DFc − log_2_FC DM| were calculated for each DEG. Each gene was assigned to the stress with the smaller difference, and a colour‐coded scatterplot was generated in R studio (‘ggplot2') to visualize the similarity. This identified whether a gene's expression under combined stress resembled one of the individual stresses more closely.

### 2.15 Multiple Linear Regression (MLR) Analysis

MLR models were defined to predict gene regulation under combination stress based on the assumption of additive effects from individual stresses at the level of gene‐wise fold changes, as observed in this study and previously (Hoheneder et al. 2023). The model was complemented with a two‐way interaction term between single stresses, accounting joint effects on gene expression under stress‐combination, which improved the prediction accuracy.

Gene expression datasets were compiled for: all regulated DEGs (*n* = 15,094), significantly regulated DEGs under combined stress per genotype (*n* = 918–4704), and DEGs significantly regulated in at least one genotype (*n* = 6558). For each DEG, average log_2_FC under combined stress was predicted from the averages under individual stresses. Across datasets and models, multicollinearity was tested between independent variables, indicating the degree to which the variances in the regression estimates are increased due to high correlation between independent variables. Variance inflation factors remained low (< 3.5) among the tested models and datasets. Model selections were based on the goodness of fit (*R* ^2^) and Akaike information criterion as an estimator of prediction error of different models. Homoscedasticity was checked in residual plots. Quantile‐quantile plots were generated to graph the distributions of residuals. Normality was not strictly required, as the analysis involved large gene expression datasets and linear models are robust to mild distributional deviations under the Central Limit Theorem (Lumley et al. 2002). Differences between *R* ^2^ and adjusted *R* ^2^ values were compared, indicating model overfitting. The regression model maximizing *R* ^2^ and minimizing the root mean square error has the following equation:

$$y\left(\mathrm{DFc}\right)=\beta_{0}+\beta_{1}\left(\text{WFc}\right)+\beta_{2}\left(\mathrm{DM}\right)+\beta_{3}\left(\mathrm{WFc}\times \mathrm{DM}\right),$$

where *y*(DFc) is the predicted expression under combined stress; *β*_0_ the intercept; *β*₁(WFc) the effect of infection; *β*₂(DM) the effect of drought; and *β*₃(WFc × DM) is the interaction between infection and drought.

Regression coefficients (*β*_0_–*β*₃) and corresponding *p* values were extracted to estimate the contribution of each predictor to gene expression under combined stress.

## 3 Results

### 3.1 Drought Stress Supports FHB Disease in Moderately Resistant Cultivars

**Figure 1:**
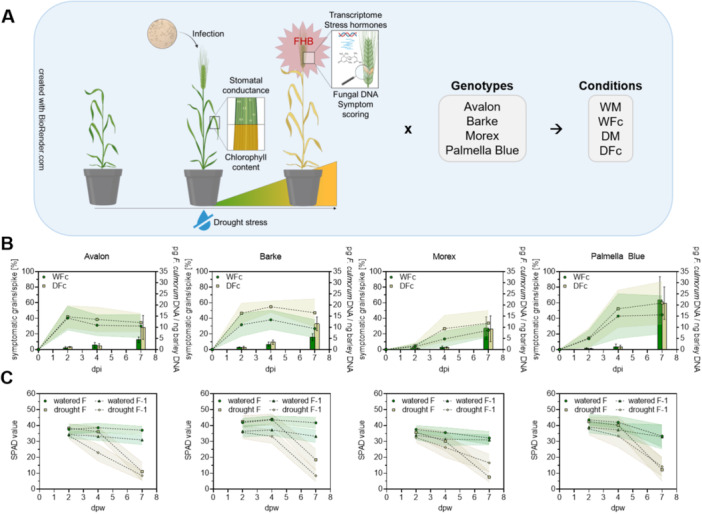
Genotype‐specific responses to drought and infection. (A) Barley cultivars Avalon (Av), Barke (Ba), Morex (Mo) and Palmella Blue (Pb) were grown under controlled greenhouse conditions. Drought stress was applied by stopping irrigation at mid‐anthesis. Plants were either watered (W) or drought‐stressed (D) and inoculated with *F. culmorum* (Fc) or mock (M). Disease symptoms were recorded at 2, 4 and 7 dpi; spikes were collected for fungal DNA, transcriptome and hormone analyses. Leaf chlorophyll was measured to monitor ripening and physiological responses. (B) FHB severity (% symptomatic grains per spike) and fungal DNA contents. Progression curves show symptom means; shaded areas indicate SD. Bars display mean fungal DNA at 2, 4 and 7 dpi with SD. (C) Mean SPAD (*n* = 18, F and F‐1 leaves) from 2 to 7 dpw in watered and drought‐stressed plants; shaded areas show SD. Differences between treatment groups were tested using Mann–Whitney *U*‐test (Table S1–S3). Created in BioRender. F. Hoheneder (2026) https://BioRender.com/wq67tos.

To better understand the quantitative FHB resistance of barley under complex stress, the present study determined the FHB severity and responses under simultaneous drought stress and infection by *F. culmorum* (DFc) in comparison to single stresses (WFc, watered *Fc*‐infected; DM, drought‐stressed mock‐infected) and non‐stressed controls (WM, watered mock‐infected; Figure 1A).

We assessed FHB severity visually and via fungal DNA quantification in the four barley cultivars (Figure 1A,B). Both watered and drought‐stressed plants showed signs of infection by 2 dpi, with increasing symptoms and fungal DNA. From 4 dpi onwards, drought stress enhanced FHB symptoms, notably in Palmella Blue, Barke, and Morex, with visual differences most pronounced at 7 dpi. Avalon showed only a slight symptom increase under drought, but fungal DNA content rose significantly (*p = 0.0286). Barke displayed a strong increase of symptoms (**p = 0.0019) and higher fungal DNA (*p = 0.0286) under drought. Morex and Palmella Blue showed no DNA differences at 7 dpi, with Palmella Blue being the most susceptible (Supporting Information S1: Tables S1 and S2).

Plants under drought conditions visually exhibited reduced turgor pressure and partial loss of green leaf area represented by the SPAD values, while the watered plants displayed normal growth and ripening. Chlorophyll contents and stomatal conductance reflected plant ripening and drought response. Irrigated plants maintained stable chlorophyll, whereas SPAD‐values of flag leaf and flag leaf‐1 decreased significantly under drought (****p < 0.001; Figure 1C), reflecting accelerated senescence. Transpiration strongly declined under drought indicated by reduced stomatal conductance (Supporting Information S1: Figure S4).

### 3.2 Plant Hormone Contents Reflect Drought, FHB Responses and Gene Expression Patterns

**Figure 2:**
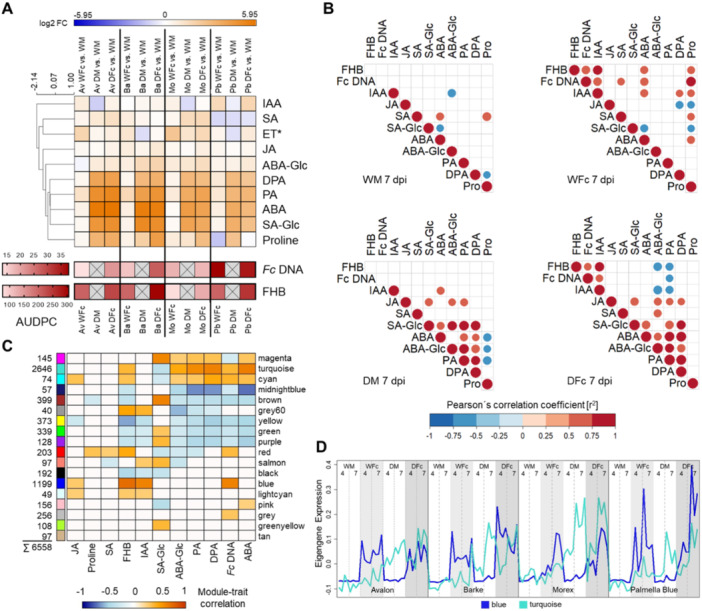
Phytohormone regulation, correlation and WGCNA in drought‐ and pathogen‐stressed barley. (A) log_2_ fold changes in phytohormone levels (AUC from 4 to 7 dpi) for WM versus WFc, DM, and DFc treatments. Hierarchical clustering highlights treatment‐specific hormone responses. The respective AUDPC values for FHB symptoms and fungal DNA indicate disease severity across cultivars. *ET: The gaseous ethylene was measured on additional spikes sampled in parallel to those used for fungal DNA and phytohormone quantification. (B) Pearson correlation matrices showing significant (*p* < 0.05) relationships among phytohormone levels, symptomatic grain proportions and fungal DNA at 7 dpi. (C) WGCNA module‐trait relationships with significant eigengene correlations (coloured boxes: FDR‐corrected *p* < 0.01) to measured stress parameters. Displayed modules contain in total 6558 genes (D) Line plots of eigengene expression profiles for the two largest modules (turquoise, blue) across genotypes, treatments and time points. [Color figure can be viewed at wileyonlinelibrary.com]

To examine pathogenesis‐related and physiological changes associated with quantitative FHB resistance, we measured hormones and metabolites by LC‐MS/MS or gas chromatography and assessed global gene expression in spikes by 3′RNA‐sequencing. Hierarchical clustering of log_2_ fold changes in phytohormone AUC values (4–7 dpi), alongside with AUDPC values for fungal DNA and visible symptoms (0–7 dpi), revealed coordinated stress responses. Pearson correlations visualized relationships between hormone levels and disease severity (Figure 2A,B). WGCNA clustered 6558 DEGs (|log_2_FC | > 1; FDR‐corrected *p* < 0.01) into 18 co‐expression modules to identify associations with measured physiological traits (Figure 2C).

Hierarchical clustering grouped several drought‐responsive hormones and metabolites with positive log_2_ fold changes under drought or combined stress. The strong positive regulation of ABA‐Glc, DPA, PA, ABA, SA‐Glc and proline confirms the successful application of drought in all four genotypes (Figures 1C and 2A; Supporting Information: Figure S4). Avalon and Barke showed strongest regulation of ABA and highest absolute ABA concentrations under drought and combination stress, indicating the strongest drought response (Figure 2A; Supporting Information S1: Figure S5). The stress hormones SA, ET and JA showed diverse patterns or slight up‐ or down‐regulation, depending on stress treatments and genotypes, while auxin showed down‐regulation under drought stress in Avalon, Morex and Palmella Blue, but up‐regulation under infection and combined stress. This was most pronounced in susceptible Palmella Blue and minimal in less susceptible Barke, which showed slightly positive fold changes of auxin across all treatments. In addition, the AUDPC integrated the progression of FHB symptoms and fungal DNA contents to display overall effects of drought or irrigation on disease severity (Figure 2A).

Pearson's correlations suggested relations between multiple stress‐associated parameters over all genotypes (Figure 2B). Under control conditions at 7 dpi, ABA showed a positive correlation with SA (*r* ^2^ = 0.60) and a negative correlation with SA‐Glc (*r* ^2^ = −0.53). Auxin negatively correlated with ABA‐Glc (*r* ^2^ = ‐0.66). Proline and SA were in a positive relationship (*r* ^2^ = 0.72), while proline and DPA were negatively correlated (*r* ^2^ = −0.52). In infected samples, auxin showed a positive correlation with the scored *Fusarium* symptoms and fungal DNA contents in WFc or DFc at 7 dpi (Figures 2B) and 4 dpi (Supporting Information S1: Figure S6). Similar correlations were also observed when all infected samples are included (WFc+DFc, 4 + 7 dpi) (Supporting Information S1: Figure S6), suggesting a strong relationship between auxin levels and FHB infection. Strong positive correlations between proline and *Fusarium* symptoms (*r* ^2^ = 0.60) or fungal DNA contents (*r* ^2^ = 0.85) were also found in watered plants at 7 dpi (Figure 2B). Auxin and ABA positively correlated under infection or drought stress alone, but this was resolved under combined stress. In line with the positive regulations of ABA, ABA‐Glc, PA and DPA under drought stress (Figure 2A), ABA and ABA‐Glc positively correlated with ABA‐catabolites PA and DPA under similar conditions (DM, DFc, 7 dpi), but not under infection stress (WFc, 7 dpi; Figure 2B). A similar pattern was found at 4 dpi (DM, DFc; Supporting Information S1: Figure S6), which confirms the close relationship of these hormones with drought.

We carried out a WGCNA to associate gene co‐expression modules with stress‐associated phytohormones and disease parameters (Figure 2C). The turquoise module revealed the strongest positive associations with ABA levels (*r* ^2^ = 0.66), DPA (*r* ^2^ = 0.64), PA (*r* ^2^ = 0.60), ABA‐glucoside (*r* ^2^ = 0.46) and with *Fusarium* symptoms (*r* ^2^ = 0.37) and fungal DNA contents (*r* ^2^ = 0.38). Its eigengene expression profile showed the strongest elevation in all drought‐stressed samples (DM) across all genotypes and for combined stress (DFc) samples of Avalon and Morex, supporting drought as the primary driver of this module (Figure 2D). GO analysis of the 2646 DEGs in this module revealed significant enrichment of drought‐ and stress‐related BPs, including ‘carbohydrate metabolic process’, ‘catabolic process', ‘response to water', ‘response to acid chemical', ‘response to water deprivation' and ‘response to endogenous stimulus' as well as MFs related to hydrolase and phosphatase activity and nutrient reservoir activity indicating stress responses, physiological adaptation and signal regulation (Supporting Information S1: Figure S7). Consistently, the turquoise module contains numerous stress‐ and maturity‐related functions, including peroxidases, ABA‐associated proteins, dehydration‐ and senescence‐related genes, late embryogenesis abundant proteins and seed maturation genes (Supporting Information S7: Dataset W17: WGCNA modules and eigengene data; turquoise module), identifying it as a large drought‐responsive gene cluster, reflecting stress‐accelerated ripening and ABA regulation (Figure 2B).

The second largest blue module (1199 DEGs) showed a strong positive correlation with FHB symptoms (*r* ^2^ = 0.74), fungal DNA (*r* ^2^ = 0.64), auxin (*r* ^2^ = 0.63) and to a lesser extent with JA (*r* ^2^ = 0.27). Its eigengene expression profile showed strong up‐regulation in the infected (WFc, DFc) samples across all cultivars, indicating a close association with infection stress (Figure 2D). GO analysis identified ‘glutathione metabolic process' as the most significant BP, and glutathione transferase activity, transferase and glycosyltransferase activity as the most enriched MFs (Supporting Information S1: Figure S8). Most DEGs in this module were specifically up‐regulated by infection or double stress, but not by drought alone (Supporting Information S7: Dataset W2: WGCNA modules and eigengene data; blue module). Notable genes included typical infection‐responsive genes with high gene‐trait associations (Supporting Information S7: Dataset W2: WGCNA modules and eigengene data; blue module). Prominent examples are a disease resistance protein (TIR‐only class, HORVU.MOREX.r3.2HG0134190) and 33 ‘Glutathione *S*‐transferases' (e.g., HORVU.MOREX.r3.5HG0424890; HORVU.MOREX.r3.7HG0665160) involved in deoxynivalenol (DON)‐detoxification (Gardiner et al. 2010). In addition, the DON‐detoxifying UDP‐glycosyltransferase *Hv*UGT13248 (HORVU.MOREX.r3.5HG0464880) (Schweiger et al. 2010; Mandalà et al. 2019) was highly expressed under infection and combination stress, but not under drought. *Hv*UGT13248 was previously identified as a highly *Fusarium*‐responsive hub‐gene with a stable expression under infection and drought‐pre‐stressed barley infection (Hoheneder et al. 2023). In addition, eight ‘2‐oxoglutarate (2OG) and Fe(II)‐dependent oxygenase superfamily proteins', putative immune regulators facilitating *Fusarium* susceptibility (Low et al. 2020), and multiple defence related genes (PR proteins, glucanases, chitinases, thaumatin‐like proteins and peroxidases) were strongly up‐regulated under infection (WFc, DFc) displaying high basal defence activity (Supporting Information S7: Dataset W2: WGCNA modules and eigengene data; blue module).

The brown module (399 DEGs) positively correlated with SA‐glucoside and negatively with fungal DNA, FHB symptoms, ABA and its derivatives (ABA‐Glc, PA, DPA), and proline, indicating a general metabolic pathogen and drought stress regulation (Figure 2C; Supporting Information S1: Figure S9). The yellow module reflects drought effects on photosynthesis and energy supply (Figure 2C; Supporting Information S1: Figure S10; Supporting Information S7: Dataset W18: WGCNA modules and eigengene data; yellow module), while the red module correlated with FHB symptoms, fungal DNA, proline and SA (Figure 2C). Magenta and cyan modules showed stress‐association patterns similar to turquoise, differing mainly in JA or SA‐Glc correlations, while midnightblue displayed opposite trends (Figure 2C). The magenta eigengene expression further distinguished Avalon and Barke from Morex and Palmella Blue, reflecting genotype‐specific regulation (Supporting Information S1: Figure S11).

Taken together, the different modules indicate that barley employs diverse co‐regulated gene sets with distinct biological functions to regulate complex stresses, each significantly linked to stress‐specific hormonal regulations or disease severity.

### 3.3 Drought and FHB Additively Contribute to Combined Stress Responses

**Figure 3:**
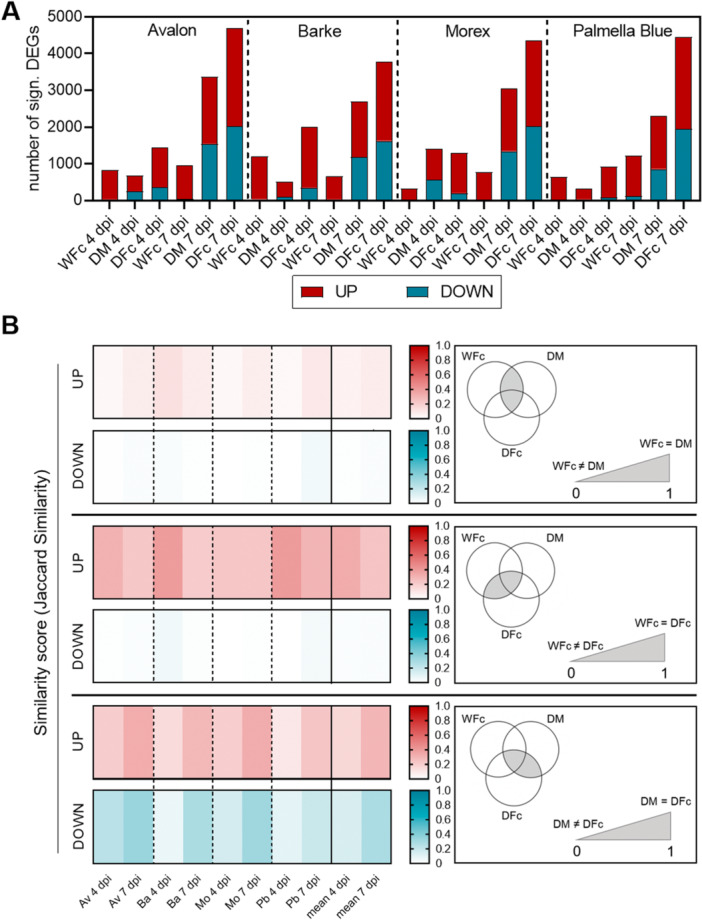
Significant DEG numbers and similarity scores between gene sets regulated under single or combination stress. (A) Significantly regulated DEGs (FDR‐corrected *p* < 0.01) across genotypes and conditions at 4 and 7 dpi. Red bars: up‐regulated; blue bars: down‐regulated DEGs. (B) Similarity scores between DEGs under single and combined stress across genotypes and time points, calculated separately for up‐ and down‐regulated genes. Venn diagrams illustrate overlapping gene sets corresponding to the similarity scores. [Color figure can be viewed at wileyonlinelibrary.com]

We compared global gene expression responses under individual and combined stress across cultivars and time points (Figure 3A; Supporting Information S1: Figure S2). DEG comparisons using the Jaccard Index (Figure 3B) revealed quantitative differences between treatments, with combined stress inducing the highest number of DEGs (4 dpi: 918–1454; 7 dpi: 3780–4704). This reflected stress severity indicated by increasing fungal DNA and ABA levels (Figures 1B and 2A). Drought alone induced the second highest DEG numbers at 7 dpi (2299–3363), increasing from 4 dpi (317–1405) across cultivars.

Across treatments, more genes were up‐ than down‐regulated with infection alone inducing several hundred up‐regulated DEGs per cultivar, but relatively few down‐regulated DEGs (max. 113 in Pb at 7 dpi). In contrast, drought and combined stress caused substantial down‐regulation, increasing from 4 dpi (DM: 31–559; DFc: 81–357) to 7 dpi (DM: 855–1541; DFc: 1624–2019), accounting for nearly half of total DEGs.

Combined stress largely followed an additive pattern: number of up‐regulated DEGs largely matched the sum of single stress DEGs, while numbers of down‐regulated DEGs exceeded pure additive effects, except for Morex at 4 dpi (Figure 3A; Supporting Information S1: Figure S2). This may be because DEG numbers are *p* value threshold‐dependent. This can cause an underestimation of single stress contributions, if non‐significant changes under single stresses add up to stronger and statistically significant changes under combined stress (see below). DEG similarity between infection and drought stress remained low (Figure 3B), indicating separated transcriptional responses.

Infection and combined stress shared on average 29% of up‐regulated DEGs, with overlap decreasing from 4 to 7 dpi, while overlap in down‐regulated DEGs was minimal because infection alone down‐regulated only few genes (Figure 3B). In contrast, DEG overlap between drought and combined stress increased toward 7 dpi across all cultivars, reflecting the rising number of drought‐responsive genes and their consistent expression under co‐occurring FHB‐stress (Figure 3B; Supporting Information S2: Dataset D1: DEG lists: All 15094 DEGs).

### 3.4 Core Stress Response Genes Are Stably Expressed Under Single and Combined Stress

**Figure 4:**
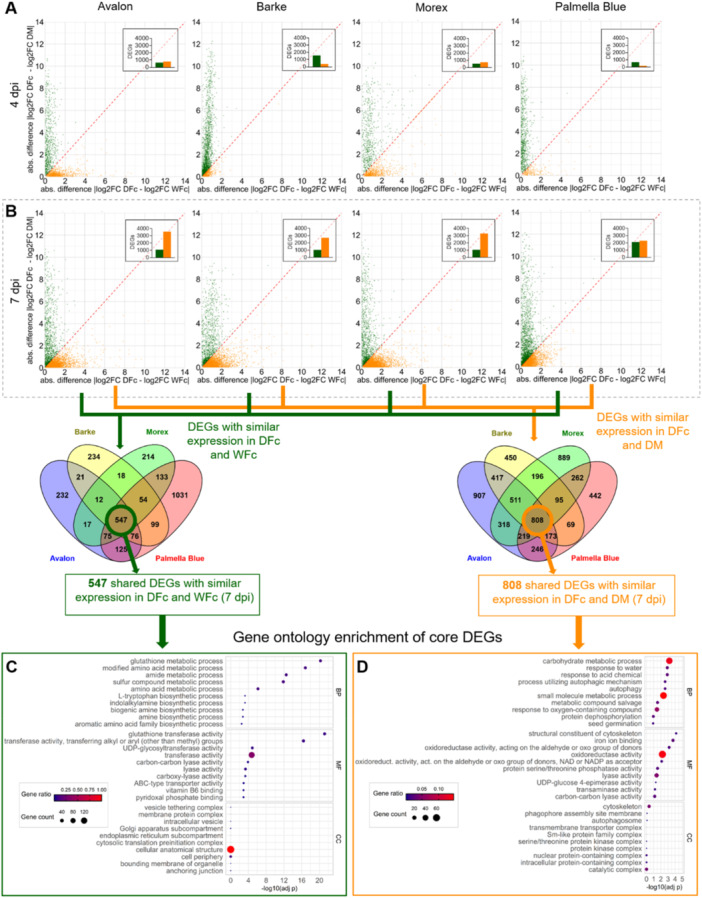
Expression similarities and GO enrichment analyses of shared core DEGs. (A) Expression similarity plots for significantly (FDR‐corrected *p* < 0.01) regulated DEGs at 4 dpi and (B) at 7 dpi. DEGs with similar expression under infection and combined stress are shown as green dots; those with similar expression under drought and combined stress are shown in orange. Bar graphs show total DEG counts. Venn diagrams show the number of shared DEGs with similar expression between DFc and WFc or DFc and DM across all four genotypes at 7 dpi. (C) GO enrichment for DEGs with similar expression in DFc and WFc. (D) GO enrichment for DEGs with similar expression under DFc and DM for biological process (BP), molecular function (MF) and chemical component (CC). [Color figure can be viewed at wileyonlinelibrary.com]

To assess how single stress responses account for gene expression under combined stress, we subtracted single stress regulation from combined stress regulation values and visualized the result for all DEGs from combined stress responses (Figure 4). In the corresponding similarity plots, DEGs near the *y*‐axis reflect infection‐like expression under combined stress, those near the *x*‐axis reflect drought‐like expression, and those near the diagonal indicate expression values under combined stress less well explainable by single stresses. At 4 dpi, many DEGs actually plot close to the *y*‐axes and thus their regulation reflects pathogenesis‐related expression patterns. At 7 dpi, more genes plot close to the diagonal and the *x*‐axis, which can be explained by additive or solely drought‐related expression patterns, likely caused by the increasing drought severity (Figure 4B). Avalon or Morex showed stronger drought effect already visible at 4 dpi (Figure 4A). Hence, infection‐responsive DEGs exhibited higher absolute differences in fold changes than drought‐related DEGs, reflecting a rapid and strong regulation upon local fungal colonization compared with the more gradual systemic drought response.

Intersecting DEGs with similar expression between DFc and WFc or DFc and DM in all genotypes revealed 547 core infection‐related and 808 drought‐related DEGs, shared across single and combined stress (Figure 4C,D). Infection‐related DEGs at 4 or 7 dpi, respectively, enriched GO terms for ‘sulphur compound metabolic process' and ‘glutathione metabolic process', underscoring the role of glutathione *S*‐transferase metabolism in detoxification during *Fusarium*‐ or DON‐induced stress (Gardiner et al. 2010; Gullner et al. 2018) (Figure 4C; Supporting Information S1: Figure S12). Enriched MFs included ‘transferase activity', ‘glutathione transferase activity', and ‘UDP‐glycosyltransferase activity', while ‘l‐tryptophan biosynthetic process' supports the role of tryptophan‐derived defence pathway in barley (Ishihara et al. 2017; Powell et al. 2017; Hein et al. 2025) and its involvement in auxin biosynthesis linked to *Fusarium*‐susceptibility (Brauer et al. 2019; Su, Zhao, et al. 2021). Highly expressed genes in these DEG sets include ‘glutathione *S*‐transferases', ‘tryptophan decarboxylases', ‘Fusarium resistance orphan protein' and ‘disease resistance protein' (Supporting Information S6: Dataset E1‐2: Shared DEGs Expression similarity).

Among DEGs with similar expression under DM and DFc (51 at 4 dpi; 808 at 7 dpi; Supporting Information S6: Dataset E3–E4: Shared DEGs Expression similarity), many were linked to drought, senescence and maturation (e.g., ‘dehydrins', ‘late embryogenesis abundant proteins' and ‘ripening‐related proteins'). GO enrichment indicated early signalling‐related processes at 4 dpi (‘dephosphorylation', ‘phosphatase activity'; Supporting Information S1: Figure S12) (Yuan et al. 2016; Zhang et al. 2023) and a shift towards drought stress adaptation mechanisms including ‘response to water', ‘response to oxygen‐containing compound' and ‘autophagy' (Liu et al. 2009; Tang and Bassham 2022) at 7 dpi (Figure 4D).

These similarly regulated genes thus represent core stress‐responsive sets across diverse cultivars in barley (Figure 4C,D; Supporting Information S6: Dataset E1–4: Shared DEGs Expression similarity).

### 3.5 Drought Response Limits FHB‐Related Gene Up‐Regulation

**Figure 5:**
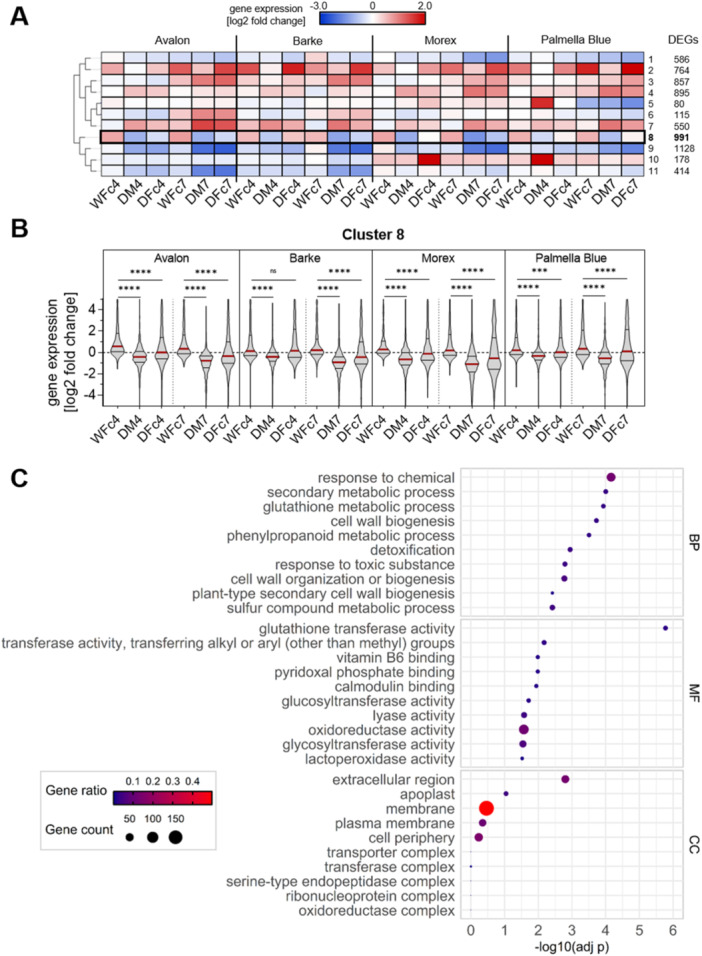
DEG and GO enrichment analyses targeting *Fusarium*‐responsive DEGs under infection stress and weaker regulation under combination stress. (A) 6556 significantly (FDR‐corrected *p* < 0.01) regulated DEGs with |log_2_FC| > 1 sorted by a self‐organizing tree algorithm (SOTA) into 11 clusters with a similar expression pattern. The heat map displays the mean log_2_ fold changes across all genotypes, treatments and time points for each cluster. (B) Violin plots represent the frequency distribution of DEGs per treatment, time point and genotype found in cluster 8 according to their gene expression magnitude. Violins show the median (red solid line) and upper and lower quartile (black lines). For better visualization, the data within a range of |log_2_FC | < 5 is shown. Significant differences between medians were tested with Kruskal–Wallis test and Dunn's multiple comparisons test at a significance level of 0.05. (ns = not significant; ***p < 0.001; ****p < 0.0001). (C) GO enrichment for DEGs found in cluster 8 for biological process (BP), molecular function (MF) and chemical compound (CC). GO enrichment analyses of the remaining clusters are shown in Supportimg Information S1: Figures S13–S22. [Color figure can be viewed at wileyonlinelibrary.com]

Overall, DEG profiles under combined stress appeared largely additive, but this does not readily explain the drought‐induced increase in FHB severity. Many *Fusarium*‐responsive DEGs were up‐regulated under infection but down‐regulated under drought, suggesting drought‐attenuated defence responses. To capture such patterns, we applied a self‐organizing tree algorithm, clustering 6558 significant DEGs (|log_2_FC | > 1; FDR‐corrected *p* < 0.01; Supporting Information S2: Dataset D2: DEG lists; 6558 sign DEGs) across cultivars, conditions and time points into 11 co‐expression groups (Figure 5A). Cluster 8 comprised 991 DEGs consistently induced by infection but suppressed under drought and largely reduced under combined stress, except in Barke at 4 dpi (Figure 5A,B). This cluster included stress‐related glycosyltransferases, glutathione S‐transferases, tryptophan decarboxylases and pathogenesis‐related proteins (Supporting Information S5: Dataset S8: SOTA clusters; SOTA cluster 8), with GO enrichment for ‘metabolism', ‘secondary metabolic processes', ‘glutathione transferase activity', ‘detoxification' and ‘cell wall biogenesis'. The attenuation of those infection‐related responses likely contributes to increased susceptibility of Avalon and Barke under drought (Figure 5c).

### 3.6 MLR Model Explains Combined Stress Responses From Single Stress Responses

**Figure 6:**
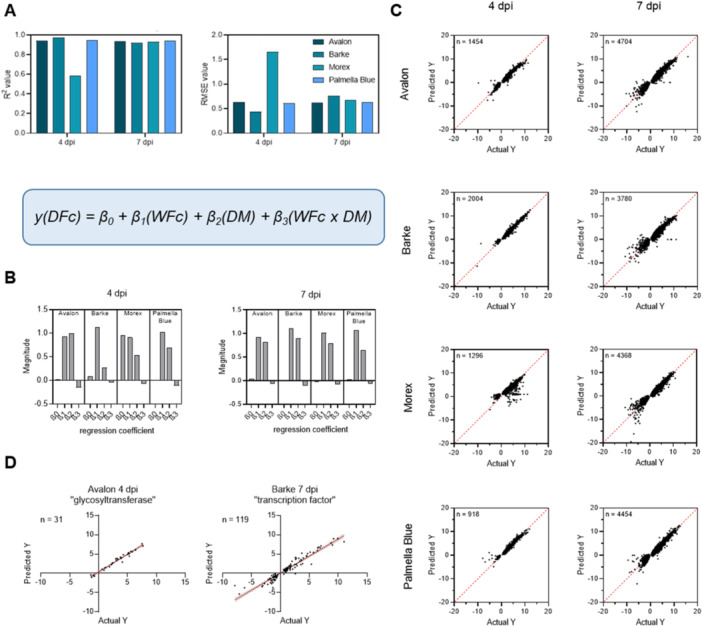
Multiple Linear Regression analysis for prediction of gene expression under combined stress. (A) Goodness of fit (*R* ^2^) and respective root mean squared error obtained from the MLR model for significantly (FDR‐corrected *p* < 0.01) regulated DEGs under combination stress per cultivar. The MLR model equation is given in the box. (B) Magnitude of regression coefficients for *β*_0_, *β*_1_ (main effect: WFc), *β*_2_ (main effect: DM) and *β*_3_ (interaction between WFc and DM) per cultivar and time point post‐infection. (C) Actual and predicted log_2_ fold changes for significantly regulated DEGs in DFc obtained from the model for each cultivar and the two‐time points post‐infection. The dashed red line shows the line of identity. (D) Actual and predicted log_2_ fold changes in combination stress for exemplary gene sets selected by keyword search in the obtained DEG lists (Supporting Information S3: Dataset M1‐8: MLR results) for ‘glycosyltransferase' in Avalon 4 dpi and for ‘transcription factor' in Barke 7 dpi. The red solid line displays the regression line and dashed lines show the 95% confidence bands for the best‐fit line. [Color figure can be viewed at wileyonlinelibrary.com]

Since the number of DEGs and the magnitude of fold changes increased with stress intensity and under combined stress, our study and previous data (Hoheneder et al. 2023) suggested that combined stress responses may be to a good part explained by the accumulation of single stress effects (Figure 3A,C). To assess additivity at the level of regulation of genes, we applied an MLR model to predict gene expression under combined stress from single infection and drought responses (Figure 6).

We used different DEG sets to optimize model performance. While predictions based on all DEGs or DEGs significant in at least one genotype were acceptable, the best fit was achieved when focusing on DEGs significant under combined stress (DFc; FDR‐corrected *p* < 0.01). The final model achieved high goodness of fit (*R*² = 0.58–0.98; RMSE = 0.44–1.65) across genotypes (Figure 6A; Supporting Information S3: Dataset M9: MLR results; Model statistics), showing that expression level under combined stress can be well predicted from single stress responses.

Very high *R*² values were observed for Barke at 4 dpi (0.98) and all genotypes at 7 dpi (0.92–0.94). RMSE values remained below 0.76 except for Morex at 4 dpi, which showed lower accuracy (*R*² = 0.58; RMSE = 1.65) (Figure 6A). Regression coefficients indicated strong positive effects of infection (*β*₁) and drought (*β*₂) on gene expression under combined stress. *β*₁ was high in Barke, Morex, and Palmella Blue at 4 dpi and in all cultivars at 7 dpi, while *β*₂ was greater than *β*₁ only in Avalon at 4 dpi and increased overall at 7 dpi, highlighting stronger drought influence at later times (Figure 6B). All coefficients were significant (*p* < 0.0001), although *β*_0_ was notably high in Morex at 4 dpi, reflecting lower model fit. Notably, *β*_3_ (WFc × DM) remained constantly low, indicating that combined stress responses are driven mainly by the additive effects of single stresses rather than by their interaction (Figure 6B; Supporting Information S3: Dataset M9: MLR results; Model statistics).

The MLR model‐predicted and measured log_2_ fold changes of DFc DEGs closely aligned with the identity line, confirming good model performance except for some deviations in Morex at 4 dpi (Figure 6C). Exemplary regression predictions for selected gene sets, such as ‘glycosyltransferase' in Avalon and ‘transcription factor' in Barke, showed tight clustering around the regression line (Figure 6D). When we applied that model separately only on genes that were down‐regulated under combined stress at 7 dpi (1624–2019 DEGs), model performance was similar to that of all significant DFc DEGs with a somewhat weaker *R* ^2^ (Supporting Information S1: Figure S23). Similarly, good predictive power was obtained for SOTA cluster 8, supporting additivity of fold changes in gene expression even if single stresses cause opposite effects (Supporting Information S1: Figure S24).

We applied the same model to published transcriptome data from a different experimental setup on *Fusarium*‐infected and 7‐day drought pre‐stressed barley (Hoheneder et al. 2023). This yielded similarly high goodness‐of‐fit (*R*² = 0.90–0.97) and low RMSE (0.44–1.01), demonstrating robustness of the MLR model across cultivars and independent combined stress experiments (Supporting Information S1: Figure S1: S25).

## 4 Discussion

The stress severity affecting crops is gradually rising with more frequent extreme weather events due to climate change (Heino et al. 2023; Martin et al. 2025). Additionally, pathogen abundance and disease severity may increase under these conditions (Delgado‐Baquerizo et al. 2020). Consequently, plants are more often exposed to combined abiotic and biotic stresses. We assessed FHB severity and global gene expression responses in different barley genotypes under simultaneous drought stress and infection. Drought stress before infection reduced FHB severity, likely due to accelerated ripening (Hoheneder et al. 2023). However, the complexity of plant stress regulations and the final outcome under combined stress cannot be generalized from individual experiments and largely depends on the genotype, stress severity, and the timing and order of the stress applications (Suzuki et al. 2014; Zandalinas et al. 2021). This study shows that simultaneous drought and *Fusarium* infection increase FHB severity, but this effect depended on the plant genotype. Transcriptome analysis revealed a largely additive regulatory response under combined stress, with increasing drought‐related effects dominating under prolonged drought. Stress hormone profiling found associations with disease parameters and gene co‐expression modules suggesting ABA and auxin regulation play important roles in limiting basal FHB resistance. A group of *Fusarium*‐responsive genes showed trade‐off effects under simultaneous drought (Figure 5), which potentially limited FHB resistance.

Our data show increased FHB severity in the two more resistant genotypes under drought. Barley regulated the highest number of genes under combined stress, indicating high physiological costs in complex stress situations (Figures 2A,C and 3A). Hence, quantitative resistance might have been only partially functional under simultaneous drought stress (Figure 1B). Differences in flowering times of 1 week between Avalon/Barke and Morex/Palmella Blue prevented simultaneous inoculation, thus limiting the full scope for data comparison. Nevertheless, all genotypes showed similar FHB severity at 4 dpi. By 7 dpi under drought, the modern varieties Avalon and Barke had lost part of their basal FHB resistance but did not become fully susceptible when compared to highly susceptible Palmella Blue. The drought‐induced ABA levels were highest in these two genotypes (Supporting Information: Figure S5), thus they strongly reacted to drought. ABA was previously shown to promote FHB severity by interfering with defence signalling (Qi et al. 2016; Buhrow et al. 2021). However, ABA positively correlated with disease parameters under infection (Figure 2B), but not under combined stress, likely because the upfront more susceptible varieties accumulated less ABA. Under double stress, ABA‐glucoside and PA showed negative correlations with FHB. In contrast, the more *Fusarium*‐susceptible cultivars Morex and Palmella Blue (Hoheneder et al. 2022, 2023) were strongly infected under irrigation, and drought did not further increase FHB severity. These genotypes accumulated less ABA under drought, likely limiting negative effects on basal resistance. Alternatively, environmental conditions generally cannot further enhance very high susceptibility to FHB. Furthermore, we found several ABA‐related and positively co‐upregulated ABA‐metabolites (ABA‐Glc, PA, DPA) and SA‐glucoside in drought‐stressed samples (Figure 2A,B; Supporting Information S1: Figure S5), which could have influenced resistance, but their exact role in FHB in barley is unclear and needs future investigation. Together, our data suggest an association of ABA with drought‐supported FHB susceptibility during ongoing pathogenesis in Avalon and Barke.

Importantly, the two largest WGCNA gene co‐expression modules (Figure 2C, blue and turquoise) each associated with respective infection‐ or drought‐stress‐related stress parameters, respectively. Similarly, examining also smaller modules and stress‐ or genotype‐specific gene clusters enabled us to describe in detail the drought, infection and combination stress responses and link gene sets to measured physiological responses (Figure 2C; Supporting Information S1: Figures S7–S10, S26–S39; Dataset W1‐20: WGCNA modules and eigengene data). Overall, association of DEG modules and hormone data provides a detailed insight into barley's response to combined stress, but does not explain increased FHB severity under drought in Avalon and Barke. Therefore, we required a detailed analysis of differentially gene expression patterns.

The overall transcriptional response shows that barley regulated independent sets of pathogen‐ or drought stress‐responsive genes (Figure 3A), which added up in numbers during the double stress response (Figure 3A). The additivity for significantly up‐regulated genes under combined stress appears clearly fulfilled, but less so for down‐regulated genes, where many genes not significantly regulated under single stresses become significant under combined stress (Figure 3B). The similarity scores reveal that the single stress responses show only a small overlap with each other and are thus clearly separated (Figure 3B), whereas WFc and DFc show a large overlap, especially in up‐regulated DEGs. This similarity decreased at 7 dpi, likely due to the increasing drought impact, which causes partially opposite regulation to the infection effect (see below). Infection mainly caused an up‐regulation of DEGs, and was partially maintained under DFc, with a clear addition of the drought response. By contrast, the overlap of up‐ or down‐regulated DEGs in DM and DFc increased between 4 and 7 dpi, reflecting the increasing drought dominance. This manifests also for down‐regulated genes in this comparison, because barley significantly regulates many more genes down under drought than infection (Figure 3A,B). This suggests that under drought stress, which affects the entire plant, many biological functions are downregulated, whereas local infection response mainly causes an up‐regulation of DEGs. Drought stress strongly affected energy metabolism (yellow module; Supporting Information S1: Figure S10) and enriched DEGs with enzyme activities involved in stress signalling and adaptation (turquoise module; Supporting Information S1: Figure S7) (Schweighofer et al. 2004; Fu et al. 2024). Infection was strongly associated with transferase activities and secondary metabolism (blue module; Supporting Information S1: Figure S8), representing a core metabolic response against *Fusarium* and its toxins (Bethke et al. 2023; Gardiner et al. 2010; Hein et al. 2025).

We further looked into gene expression patterns that illustrate the individual stress responses and dynamics of regulation (Figure 4A,B). This allowed us to split DEGs in those with similar expression between DFc and WFc or between DFc and DM (Figure 4A,B). Such genes, similarly regulated across all genotypes, show clearly separable enriched biological functions important for pathogen defence or drought stress mitigation. Interestingly, these genotype‐spanning ‘core DEG sets’ still consist of a few hundred genes (Figure 4B–D). Here, the DEGs involved in pathogen defence again show strong enrichment in transferase activities, including glutathione‐*S*‐transferases and UDP‐glycosyltransferases (Figure 4C). This supports that oxidative stress balance and DON detoxification are important against *Fusarium* and are maintained even under combination stress. Notably, a recent study found in a wild barley accession several UDP‐glycosyltransferases as crucial in drought tolerance by modulating hormone homoeostasis and secondary metabolism (Feng et al. 2024). It needs to be analysed which individual UDP‐glycosyltransferase might fulfil a double function in pathogen and drought stress resistance. ‘Core DEGs’ show an overall up‐regulation during infection, often down‐regulation under drought, but again up‐regulation under combined stress. Among the 6558 significantly regulated DEGs, many pathogen‐induced DEGs show similar fold changes in WFc and DFc (e.g., cluster 2 in Figure 5; Supporting Information S2: Dataset D2: DEG lists; 6558 sign DEGs). Conversely, we found cluster 8 of 991 infection‐related DEGs showing mostly attenuated or opposite regulation under combined stress compared to infection stress alone (Figure 5; Supporting Information S5: Dataset S8: SOTA cluster; SOTA cluster 8). This includes again UDP‐glycosyltransferases, glutathione *S*‐transferases, tryptophan decarboxylases, pathogenesis‐related proteins and enriched functions in cell‐wall biogenesis, together likely important for mycotoxin detoxification and basal defence against FHB (Kang and Buchenauer 2002; Boddu et al. 2006; Gardiner et al. 2010; Tucker et al. 2021; Bethke et al. 2023). This suggests an additive but contrasting regulation of DEGs in this cluster, which could reflect a trade‐off between *Fusarium* and drought responses. It further shows that drought can compromise infection‐induced defence outputs by weakening expression of infection‐responsive genes, thus supporting fungal success.

The attenuated expression of larger groups of defence genes implies a physiological network sensitive to additional drought stress. This may partially explain our findings, but it is likely that under double stress, defence was limited or superimposed at several levels. It is also possible that defence gene expression was functional, but simultaneous drought may have partially compromised the translation of defence genes into proteins. This could be explained by strong alterations of the translational machinery under drought (Lei et al. 2015) and likely a suppressive role of ABA on global translation efficiency, as reported for *Arabidopsis thaliana* (Zhang et al. 2025). As shown for differently drought‐tolerant rice genotypes, sensitive plants show reduced translation under drought (Dawane et al. 2024). We speculate that in combination of drought with the fungal protein translation inhibitor DON, the moderately resistant varieties were restricted in their *Fusarium* response, whereas susceptibility did not further increase in the already very susceptible genotypes. This suggests studying the impact of drought on translation level of defence genes decisive for FHB resistance.

Drought stress can also limit *Fusarium* success in barley ears, when applied before infection, which might limit fungal growth by accelerated ripening and grain maturity (Hoheneder et al. 2023). In contrast, the simultaneous onset of the two stresses may have led to conflicting responses that require a prioritization of stress responses and partial suppression of the pathogen response, which led to a better establishment of the fungus. For example, quantitatively resistant Barke (Hoheneder et al. 2022, 2023) showed a strong and early response to the fungus through regulation of many genes under both WFc and DFc conditions at 4 dpi (Figure 4A), but the regulation subsequently shifted towards a more drought‐responsive pattern at 7 dpi, similar to the other genotypes (Figure 4B). Drought might have hampered maintenance of this strong and early defence response of Barke thus supporting FHB. Drought negatively affected photosynthesis and energy supply as indicated by GO enrichment, for example, in the yellow module (Supporting Information S1: Figure S10) that showed negative correlations with several up‐regulated drought stress‐related phytohormones (Figure 2C). Thus, despite a clear *Fusarium* response, further signal processing was potentially not successfully implemented into effective defence due to a lack of energy and resources or contradictory stress signals under additional drought. This suggests that breeding for drought tolerance might indirectly protect disease resistance by warranting physiological resources for defence.

When barley is facing combined *Fusarium* and drought stress, its response is largely composed of single stresses and lacks a unique combination stress response (Hoheneder et al. 2023). This modularity is evident in the global DEG counts and overlaps (Figure 3A,B) and in expression similarity plots (Figure 4A,B). Although drought and infection DEG sets show little overlap, both align with combined stress response, indicating integration of largely independent drought‐ and infection‐responsive modules across cultivars (Figure 3B). Combined abiotic and biotic responses can induce unique transcriptional responses (Gupta et al. 2016; Zandalinas et al. 2021), but we successfully applied an additive MLR model to predict gene‐wise expression under combined stress using two independent experiments (Figure 6; Supporting Information S1: Figure S25; Hoheneder et al. 2023). The MLR analysis confirmed that gene expression responses under combined stress largely reflect additive contributions of single stresses, with minor influence of interactions terms. This is notable because very different physiological and hormonal signalling pathways are activated by drought and *Fusarium* infections, affecting, for example, ABA accumulation, storage and desiccation responses, photosynthesis and transferase activity (Supporting Information S1: Figure S40). It would be interesting to compare this to combinations of physiologically more similar stresses.

The ability to reconstruct combined stress responses from single stress expression profiles suggests that, at least for barley, transcriptional regulation under combined stress can be approximated as an overlay of largely independent stress‐response modules. A similar modular organization has been reported in model plants (Tan et al. 2023), but this hypothesis requires validation in meta‐analyses using barley transcriptomic data from additional stress combination experiments. Interestingly, the timing of the drought application appears to influence FHB severity rather than the additivity of transcriptional regulation (compare this study Figure 6, Supporting Information S1: S25; Hoheneder et al. 2023). Although this requires experimental validation across further stress combinations, our results indicate that predictive estimation of barley stress responses at the single‐gene level is feasible and may become increasingly robust as more combination‐stress datasets become available. This has important implications for understanding and predicting stress adaptation and for breeding barley varieties that combine drought tolerance and pathogen resistance. Notably, together with our previous study on early‐applied drought stress (Hoheneder et al. 2023), our data suggest that *F. culmorum* can only profit from contrasting regulatory responses in barley head tissues as long as ripening and senescence have not progressed too much.

## 5 Conclusions

Barley responses to simultaneous *Fusarium* infection and drought stress are largely additive and mainly consist of responses to each single stress. Although combined stress resulted in more significantly regulated DEGs than single stresses alone, predictive gene‐wise regression modelling demonstrated that expression under combined stress can be reconstructed from single stress responses, indicating additive regulation rather than a stress combination‐specific genetic program. This reflects modular stress regulation, in which independent drought‐ and infection‐responsive gene modules together explain most of the combined stress response. While common DEG sets highlight robust defence and drought adaptation functions, we also identified a large infection‐related gene cluster with lower expression under combined stress. This attenuation of the pathogen response, together with genotype‐specific hormonal shifts such as elevated ABA in Avalon and Barke, likely explains drought‐increased FHB in these cultivars. Nevertheless, the tested genotypes partially maintained quantitative FHB resistance even under drought, suggesting breeding potential for stacked drought tolerance and FHB resistance. The modularity of barley's stress responses, together with genotype‐dependent defence constraints, provides new insights and is relevant for breeding robust and stress adapted cereal crops.

## Conflicts of Interest

The authors declare no conflicts of interest.

## Supporting information

Supporting file 1

Supporting file 2

Supporting file 3

Supporting file 4

Supporting file 5

Supporting file 6

Supporting file 7

## Data Availability

All data supporting the findings of this study are available throughout the paper and online accessible: Raw sequencing data has been deposited at the NCBI Gene Expression Omnibus (accession GSE310570).
